# Healthcare Providers’ Perspectives and Role in Improving Patient Engagement in HIV Treatment and Care in Almaty, Kazakhstan

**DOI:** 10.3390/ijerph182212138

**Published:** 2021-11-19

**Authors:** Elizabeth J. King, Ainur Zh. Kussainova, Jangul Erlon-Baurjan, Zhamilya S. Nugmanova

**Affiliations:** 1 Department of Health Behavior and Health Education, School of Public Health, University of Michigan, Ann Arbor, MI 48109, USA; jackieer@umich.edu; 2 Department of Epidemiology, Asfendiyarov Kazakh National Medical University, Almaty 050000, Kazakhstan; almaarasan@mail.ru; 3 Division of HIV Infection, Asfendiyarov Kazakh National Medical University, Almaty 050000, Kazakhstan; nugmanova.z@kaznmu.kz

**Keywords:** Kazakhstan, HIV/AIDS, HIV treatment, healthcare providers, patient-provider communication, qualitative research methods

## Abstract

The HIV epidemic continues to grow in Eastern Europe and Central Asia. At the same time, there are major gaps in engagement in HIV treatment and care among people living with HIV (PLHIV) in the region, including Kazakhstan. Healthcare providers may have the potential to improve patients’ initiation of and adherence to HIV treatment; however, their perspectives and experiences are often overlooked in the research from Kazakhstan. The purpose of our study was to gain an in-depth understanding of how providers perceive the factors influencing PLHIV’s engagement in HIV treatment and care and to identify opportunities for expanding the role that providers can have in improving patient initiation and adherence to treatment in Kazakhstan. Through purposive sampling, we conducted in-depth interviews with 14 healthcare providers at the City AIDS Center in Almaty. We used team-based coding and thematic analysis in order to analyze the data. Quotes from providers were translated from Kazakh and Russian to English to illustrate the themes. Our results show that providers currently view their role as the need to break down myths around ART medications, and to educate and motivate patients to accept their diagnosis and learn to live with HIV. The need to address stigma and social isolation among PLHIV, the risk of overworked providers, and the need to improve patient-provider communication strategies are areas to consider for further interventions. Patient-provider communication interventions are needed in Kazakhstan in order to help meet the country’s 90-90-90 HIV targets.

## 1. Introduction

The HIV epidemic in Eastern Europe and Central Asia (EECA) is one of the fastest growing in the world, and access to and adherence to antiretroviral treatment still requires significant work. Since 2010, new HIV infections in the region have increased 72%, and AIDS-related deaths have increased 24% [1]. In 2019, EECA had a 10.1 incidence:prevalence ratio, which surpassed any other region in the world [1]. Equally concerning is that only half of PLHIV in the region are virally suppressed [1]. While more attention has been paid to countries such as Russia and Ukraine, Central Asia has experienced an increase in the number of newly diagnosed HIV cases in the past couple of decades. For example, in Kazakhstan, there was a 106% change in new HIV infections between 2010–2017 [2]. Deaths among PLHIV in Central Asia have also increased in recent years [3].

Kazakhstan is making progress in controlling the HIV epidemic; however, more work is needed to meet the global health goals. There are an estimated 27,794 people living with HIV (PLHIV) in Kazakhstan [4]. Among them, 82% of all PLHIV are aware of their status; 68% of those diagnosed are on antiretroviral treatment (ART); and 78% have achieved viral suppression [1]. While these numbers indicate improvement, they remain below the global goal of “90-90-90” needed to end the HIV epidemic. In Almaty, the largest city in Kazakhstan, less than one-third of registered adults living with HIV are on ART [5]. Kazakhstan has stood out among other Central Asian countries as having more promising approaches in addressing the epidemic [6]. Kazakhstan recently committed to the “test and treat” approach, and now provides ART to all PLHIV regardless of clinical indicators [7]. Nonetheless, if the country is going to successfully address its HIV epidemic, then more work is needed to engage PLHIV in HIV treatment and care.

This is particularly true for key affected populations in Kazakhstan. As with much of EECA, the HIV epidemic has disproportionately burdened key affected populations, namely people who inject drugs (PWID), sex workers (SW), men who have sex with men (MSM), and incarcerated populations. There are an estimated 178,000 such key affected persons in Kazakhstan [4]. The HIV prevalence among these populations continues to rise. HIV prevalence among MSM increased from 3.6% in 2015 to 6.2% in 2018; among SW—from 1.2% in 2015 to 1.9% in 2018; and among PWID—8.3% in 2016 to 9.2%% in 2017 [4]. Any approaches to address the HIV epidemic in Kazakhstan, should take into account the need for reaching these key affected populations. This often presents challenges, including fear of stigma and discrimination, for patient-provider communication.

Most of the information about adherence to ART in Kazakhstan, and the EECA region more generally, comes from the perspective of PLHIV. While these perspectives are obviously critical, the information from healthcare providers can also be very valuable to planning interventions and implementing clinic-based programs.

Previous research from other settings has shown that patient-provider relationships have been essential in shaping patients’ behavior and attitudes towards health. Patient-provider communication has been shown to foster a more accessible and information-sharing clinical environment in which patients can make better decisions about managing their illness [8]. A study in Mali showed that effective patient-provider communication helped PLHIV to determine actual steps to overcome barriers to engaging in HIV treatment and care [9]. A study in Russia showed that nearly half of participants said positive experiences with HIV medical providers helped them to remain engaged in care [10]. Gaining an understanding of the healthcare providers’ perspectives in HIV treatment and care in Kazakhstan will provide important information for intervention planning in this setting.

Kazakhstan inherited the Soviet vertical system for providing care for people living with HIV. There is a network of AIDS Centers that focus on prevention and control of HIV starting at the national level, 14 regional centers, and three centers in the larger cities of Nur-Sultan, Almaty, and Shymkent. Medical doctors trained in infectious diseases residency, along with nurses, provide care for PLHIV who have registered at these centers. The purpose of this study was to learn the perspectives of healthcare providers at the City AIDS Center about what factors influence PLHIV’s engagement in HIV care and treatment and to identify the potential roles that healthcare providers can play in improving engagement in and adherence to HIV treatment in Almaty, Kazakhstan. The goal is to use this information to identify clinic-based and provider-initiated interventions to improve treatment outcomes for PLHIV in Almaty.

## 2. Materials and Methods

We conducted a qualitative study based at the City AIDS Center in Almaty, Kazakhstan. The Institutional Review Board at the University of Michigan and administration of the Almaty AIDS Center approved the study protocol. Data collection occurred between October and December 2018.

Healthcare providers, who have direct interaction with patients at the AIDS Center, were eligible to participate in the study. A total of 23 such healthcare providers were employed at the AIDS Center. The research team, together with the AIDS Center administrators, organized an informational meeting with healthcare providers to let them know about the study and to recruit them for an individual, in-depth interview. Providers who were interested in participating were asked to contact one of the researchers to arrange an interview time. EK and AK conducted the interviews. We obtained informed consent prior to the interview, and guaranteed participants that their participation was voluntary and confidential. We conducted the interviews in a private office space at the AIDS Center. All interviews were audio-recorded. The interviews lasted on average about 47 min. We used a semi-structured interview guide that included the following topics: description of “typical” patients, description of any difficulties experienced with patients, challenges in their work, perceptions of effective strategies they use to overcome challenges, observations of what factors influence ART adherence, recommendations for improving adherence, and reflection on additional skills or information they would like to have as providers at the AIDS Center. At the end of the interview, we asked participants to offer their own reflection on the topic of HIV treatment and care adherence. The interviews were conducted primarily in Russian, but some small parts of the discussions were held in Kazakh based on participants’ preference.

The interview recordings were transcribed verbatim in Russian. The parts of the interviews in Kazakh were also transcribed and translated into Russian for data analysis purposes (JE). Data were then imported into Dedoose qualitative data managing software [11]. We used a team-based coding approach for our thematic analysis. We collectively developed a codebook based on our research objectives and the topics included in our interview guide. Three data coders (EK, AK, and JE) used the codebook to start coding data and establish intercoder consistency. While our approach was largely deductive based on the research objective, we also used inductive coding when we noticed important information that did not fit into one of the existing codes in the codebook. After coding all the data, we read over the code reports and noted both salient themes and important ideas in the data. We met to discuss each of the code reports and through this iterative process grouped our findings into several larger themes related to patients’ adherence to ARVs and the influence of health care providers in patient adherence. The entire research team reviewed and discussed the identified themes. Selected illustrative quotes were translated from Russian into English (EK, ZS) and reviewed by all members of the multilingual research team.

## 3. Results

A total of 14 health care providers participated in the interviews. This included six infectious disease doctors, four nurses, one gynecologist, one pediatrician, one dermatologist, and one psychologist. Many of the providers we interviewed have been working at the AIDS Center for more than a decade. Responsibilities of the providers included patient education and counseling about HIV, dispensary observation, prescribing ART, treatment of opportunistic infections, as well as family planning counseling. Overall, participants shared the idea that *“I think that they [patients] always need our understanding, our empathy, our support,”* (Participant 7). The providers at the Almaty AIDS Center realize that they can play important roles in helping their patients start and adhere to ART. However, they also recognize that there are barriers to this and areas where improvements are needed. We identified several themes across our data in regard to the research objective we set out to address. These include: the need to break down myths around ART medications, education and motivating patients to accept and adjust to their diagnosis, the need to address stigma and social isolation among PLHIV, the risk of overworked providers, and the need to improve patient-provider communication.

### 3.1. Breaking down the Myths Associated with ART

One of the most important functions of healthcare providers at the AIDS Center is to supply their patients with information, answer their questions, and dispel any misinformation patients have in regard to ART. The providers discussed how the lack of correct information is one of the biggest barriers to patients starting and adhering to HIV treatment. The majority of the providers talked about how *“those patients, who do not believe their [HIV] diagnosis, are the ones who don’t want to come at all [to the AIDS Center],”* (Participant 14). Many said these patients create the most difficulty for them as HIV-care providers. *“They do not want to believe it or do not believe it. So they consider themselves to be uninfected. It is difficult to work with these patients who consider themselves to be healthy and not ill. These patients will not come for checkups, not come for tests, not take medications. And this is difficult,”* (Participant 7). Some providers talked about how times have changed and there are fewer HIV-denialists; nonetheless, *“there are irresponsible people. It is difficult to work with people who consider themselves healthy because they do not believe in their diagnosis,”* (Participant 4). Many of the providers talked about needing to spend time trying to provide information to their patients because some patients are misinformed about HIV and the opportunities for HIV treatment. Many providers noted that patients may turn to online communities that propagate the ideas of “AIDS denialism” or ART being unsafe. Therefore, providers see their role being to educate their patients in order to dispel these myths. This is especially true if patients are recently diagnosed and asymptomatic. For example, a provider will tell her patient *“while you are thinking about whether or not to take treatment, time is moving forward. The virus will not wait for you. Your immune system will not wait for you…now your immune system is good but your immune system has this virus. Then you start feeling weak, start sneezing, coughing and it is too late. Then it is tuberculosis,”* (Participant 13). The provider went on to note that *“Our patients are not dying from HIV-infection, from the virus itself. They are dying from tuberculosis.”*

### 3.2. Learning to Live with HIV: Education and Motivation

Another important role that health care providers have is to educate patients about HIV and motivate them to seek treatment. A lot of effort is given to helping patients accept their HIV diagnosis. As one provider explained: *“When I conducted [post-test] counseling, she was very hysterical. It was all very difficult,”* (Participant 5). The psychologist mentioned that *“the most difficult patients are those who fall into a deep depression after diagnosis and I cannot drag them out of this swamp,”* (Participant 4). Or a nurse who said *“We repeat this again after the doctor, because some patients can ask ten times. And we explain it ten times,”* (Participant 3). Providers realize the importance of facilitating patients through the necessary steps to accept their diagnosis and to engage in treatment and care. This is also an area that providers expressed that they would like to have more training or tools especially to help reach more difficult patients. As one provider explained:

*“I would like to know how to reach those parents who have difficulties with adherence; to learn how it is done in other countries…what else can be done in addition to repeat conversations, in addition to the work of psychologists and social workers. We involve all of these. What else can we do in these situations? How do we convince the parents? This is the main thing. And secondly, what else can we do to get pregnant women to understand that the future of their child is in their hands? …We all know perfectly well that it takes time to accept a status, and time does not wait,”*

(Participant 2)

One of the most salient examples of this is health care providers helping patients to understand that HIV is a chronic illness and that treatment had advanced immensely allowing PLHIV to live long and healthy lives. As one provider described, the best motivator for starting and adhering to ARV is *“to give them complete information, break it down and show it so that it is clear, through pictures, to show how it [ARV] works. So that a person remembers how the medicine works… and also to let them know that treatment is different today and this is also an incentive for good adherence. Detailed information is needed,”* (Participant 4). Many of the providers were able to talk about how they had successfully motivated patients to start HIV treatment through creating a trusting relationship, especially for those patients who had not disclosed their HIV status to family and friends. For example, *“[The patient] did not trust anyone, not any dissidents, not the Internet. She only talked to us. And now her health is much better because she had this close connection with us. And she tells others that she only trusts coming here [to the AIDS Center],”* (Participant 9). Getting patients to start ARV and seeing their health improve also provides great motivation for the healthcare providers in their work. As one provider shared about a patient who was very sick: *“But then she started to take treatment as prescribed. I explained everything to her. This desire to help a person comes when you see it before your eyes how she is getting better. She gained weight and got better. Now she is adherent, taking treatment, and you see that the person is better. The desire to help people and that they become healthy—this is what helps me [to do my job],”* (Participant 6).

### 3.3. Stigma and Social Isolation

One of the major barriers to engagement in ART is the widespread stigma associated with HIV in Kazakhstan. As one provider explained: *“Even when we have told them their diagnosis, we ask ‘Will you tell your relatives?’ They reply ‘Of course not. Why? Because they will kick me out of the house’,”* (Participant 3). The stigma serves as a barrier to accessing HIV care and treatment. Another provider explained, *“Many patients talk about that, that they are afraid to come to the AIDS Center because it could reveal their status. Many do not come because of this fear. The building is located right in the city center. Maybe this influences things,”* (Participant 7). For many, the stigma is intersectional. There are various forms of stigma associated with marginalized identities, such as people who inject drugs. As one provider mentioned: *“Drug users, PWID, face the biggest problems in coming to the AIDS Center. It is especially difficult for pregnant women, when they learn their diagnosis during pregnancy, for Muslims, for HIV-denialists. They don’t want to disclose,”* (Participant 14). The providers noted that patients do not want to start ART because they worry that it will disclose their status to others. For example, *“some patients don’t believe their diagnosis. Some hide it from their relatives; therefore they don’t want to take medications. For some, their husbands don’t know [about their HIV status]. For some, their wives don’t know,”* (Participant 10). The stigma and fear of disclosure also serve as a barrier for providers having in-depth discussions with patients. One provider explained how a patient *“when she comes, I quickly examine her because she is afraid to meet people she knows. While I am examining her, she is sitting there all red in the face and her blood pressure goes up. She gets her pills and quickly runs out,”* (Participant 5). Stigma also makes it difficult for providers to follow-up with their patients who miss appointments, who do not pick up their ART prescriptions, or who have completely stopped coming to the Center. Providers are aware that many of their patients *“don’t want to [take ART] because you [providers] will constantly be calling, constantly looking for me. Then all my neighbors will come to know, then my relatives who live with me. I don’t want anyone to know. So I quietly and peacefully get the pills, and then throw them away. I know that these pills will never make things better for me. I am 100% certain of that,”* (Participant 14). The providers we interviewed talked about the need to address the social stigma around HIV and many mentioned that they needed more training or guidance on counseling patients through these issues.

In part due to societal stigmatization, providers discussed how PLHIV sometimes experience social isolation and that this has a negative impact on their HIV treatment outcomes. *“There are patients, single women, single men, who are at the age to… well they don’t have a family, they don’t have children. They think ‘I am not needed by anyone. Why should I get treated?”* (Participant 14). Nearly all providers talked about how lack of support is a barrier for patients staying engaged in HIV care and treatment. When patients are so afraid to tell even their parents, relatives, and friends, they cannot receive the social support that they need. The providers we interviewed overwhelmingly agreed that addressing the social isolation many PLHIV feel could have a positive impact on engagement in HIV treatment. While there is one psychologist on staff at the AIDS Center, many of the doctors and nurses are often providing some emotional and psychological support to patients.

### 3.4. Overworked Providers

Another important consideration for the role of healthcare providers is that many report feeling overworked and overextended. Some providers shared that they are short-staffed at the clinic. *“Everything is okay except we don’t have [enough] doctors. We have to work in several sections. For example, we have to see 50-60 people a day. And for one person there is not enough time to explain things to patients,”* (Participant 6). Oftentimes the doctors are very busy and the nurses are the ones who might spend more time with the patients and provide counseling. One nurse, who explained how there are not enough doctors or nurses given the crowds of patients waiting, attributed this to the fact that *“people are afraid to come work here. As soon as they hear that it is the AIDS Center,”* (Participant 13).

Feeling overworked includes not only the volume of work and time, but also the psychological and emotional impact of their work. *“It is not so much that we get tired physically, as much as emotionally. We have to take it all in from everyone. And especially the drug users; they are energy vampires,”* (Participant 13). Some providers talked about how they have patients who have not disclosed their HIV diagnosis to anyone and only confide in the AIDS Center staff. As one provider shared: *“I can consult over the phone. Sometimes patients call in the middle of the night. Because they are afraid or have some worries. I always answer and I try to be available for them,”* (Participant 7). During clinical hours however, providers reported having *“an average of 20 min with a patient. And this is not enough because he has questions. We cannot kick him out, but on the other side of the door there are others telling us to hurry because they need to get to work. Some come after lunch when there is less of a crowd. But then we also are in a hurry because we have other work to do,”* (Participant 3).

### 3.5. Improving Patient-Provider Communication

One of the main needs that participants discussed was for interventions to improve patient-provider communication. As one provider said, *“It is difficult when they [patients] do not connect with you. When they don’t find some common ground with the doctor. For this, you need to know your patient for a long time, in order for them to gradually build some trust towards you,”* (Participant 5). The providers discussed that they do work to build these relationships, but that they would like to know more strategies in this area. This is especially true given the time and resource constraints. They would like to learn more about how to effectively communicate with some of the more difficult patients, for example HIV denialists or partners of HIV denialists, and people who use drugs and alcohol. The providers also mentioned difficulties in reaching patients who refuse to take ART due to religious reasons, patients who believe that they are fine without ART because they are asymptomatic, and patients who worry about the side effects of starting ART. Providers explained that they *“discuss the test results, explain the patients’ responsibilities and rights, We try to explain and motivate. They [patients] do not understand the first time, we have to explain again,”* (Participant 1). Many felt that they are able to build this trust with patients and that despite any difficulties, *“regardless, most patients trust their doctor the most.”* Doctors acknowledged their potential role in encouraging patients to adhere to treatment. As one explained, *“Well, first of all, the doctor should do it. It seems to me that the doctor has the main role in maintaining patients’ adherence…I guess it is not that the doctor is responsible for 100%, but at least 60-70% is the doctor’s role in this. And 30% is the role of the patient,”* (Participant 7). Most providers acknowledged that there is the need for a collective effort and that there is *“a preference to go as a team to work with patients who are not coming. We have done this many times, when it is me, a social worker*, *psychologist, NGO representative,”* (Participant 2).

The healthcare providers talked about the need for more seminars, trainings, and educational activities to improve their communication around the need to start and adhere to treatment and to address some of the challenges that they observe among their patients. For example, *“We need to address the stigma in order for patients to remain in treatment. We need to organize more frequent trainings, round tables, where the doctors of these patients attend and to participate in various role-playing activities,”* (Participant 11). Another idea was to arrange educational activities *“to let people know that we have these medications and we need to increase the communication skills of doctors. Not just sit and listen to a lecture, but somehow also involve people who would come and openly talk about their status… and seminars to show that the new medications [ARVs] are highly efficacious. These joint seminars are needed to tell about how we also have these medications,”* (Participant 1).

## 4. Discussion

We conducted this study in order to gain a more in-depth understanding of the perspectives of healthcare providers working at the AIDS Center in Almaty, Kazakhstan. Our results highlight both the barriers to ART adherence that providers identify among their patients and the role of providers in improving treatment outcomes. Acceptance of diagnosis, reliable information, stigma, and social isolation are all factors that influence engagement in HIV care and treatment. Healthcare providers in our study discussed how they work to address these issues, but also note that they would like to increase their capacity to break down the barriers that many of their patients continue to face in accessing treatment services. One potential area for expansion is improved patient-provider communication. The healthcare providers in our study talked about building trust as a key component of their work. Any recommendations to expand provider-patient communication should take into account that healthcare providers already experience being overworked and short-staffed.

Our study was important to conduct because the voices of healthcare providers in Kazakhstan, and in Central Asia more generally, have largely been lacking from the public health literature. We are not aware of other qualitative studies that have investigated the role and perspectives of healthcare providers in regard to HIV treatment and care in the country. The City AIDS Center providers’ perspectives can enable interventions focusing on social behavior change communication (SBCC) between providers and patients in this setting.

Evidence from meta-analyses and systematic reviews show that psychosocial interventions are an effective approach to improving ART adherence [12]. Formal and informal institutions play important roles in improving HIV treatment adherence [13]. Our study results are important for developing and adapting a clinic-based intervention that focuses on provider-patient communication. We learned from this study that the providers at the AIDS Center strive to meet the needs of their patients and are open to learning new ideas about effective social and behavior change communication models. Other studies have shown that by changing the style of communication to be more “patient centered” can significantly motivate PLHIV on antiretroviral therapy to openly discuss adherence problems [14]. Healthcare providers in our study discussed that it can be particularly difficult to communicate with certain populations about initiating and adhering to HIV treatment. Research from other settings has shown the provider-initiated interventions can positively influence adherence to ART among “hard-to-reach” PLHIV [15]. Healthcare providers at the City AIDS Center in Almaty are aware that this is an area that needs additional intervention efforts and are willing to learn more social and behavioral change communication strategies to reach PLHIV who are not engaged in treatment and care, including PWID.

The major strength of this study is that it provides insight on the healthcare providers’ perspective in Kazakhstan, which is often lacking in comparison to the study of the patient perspective of PLHIV. Nonetheless, there are limitations worth noting. Social desirability in reporting about their work may have influenced what providers shared with us during the interviews. We attempted to reduce this bias by ensuring that all participation was confidential and AIDS Center staff would not know who participated or not, and that the results would only be presented in the aggregate. Moreover, the two interviewers have no affiliation with the AIDS Center. Another limitation is that the study results are all self-reported, meaning that we did not observe the provider-patient interactions ourselves as researchers, but relied on the recollection of healthcare providers. An additional step in this line of research could be to include observations as a qualitative data collection tool. Additionally, the perspectives of patients would also allow for further triangulation of the data. Despite these limitations, this study provides valuable information for developing clinic-based interventions to improve HIV treatment and care among PLHIV in Almaty, Kazakhstan.

## 5. Conclusions

This study aimed to gain insight into the perspectives of healthcare providers at the City AIDS Center about what factors influence PLHIV’s engagement in HIV care and treatment and to identify the potential roles that healthcare providers can play in improving engagement in and adherence to HIV treatment in Almaty, Kazakhstan. The results from this qualitative inquiry indicate that healthcare providers can be and are willing to be instrumental in motivating and educating patients to start and adhere to treatment. Healthcare providers play an important role and should be considered an important component in designing and implementing interventions to improve treatment outcomes among PLHIV in Kazakhstan. Focusing interventions on SBCC through training healthcare providers is an important area of for expansion in order to improve initiation of and adherence to ART in Kazakhstan.

## Data Availability

The data presented in this study are available on request from the corresponding author. The data are not publicly available due to the confidentiality ensured to participants.
